# Supplementary figures and images for: Metabolomic/lipidomic profiling of COVID-19 and individual response to tocilizumab
Source: PLoS Pathog. 2021 Feb 1;17(2):e1009243. doi: 10.1371/journal.ppat.1009243 (PMC7877736; doi:10.1371/journal.ppat.1009243)

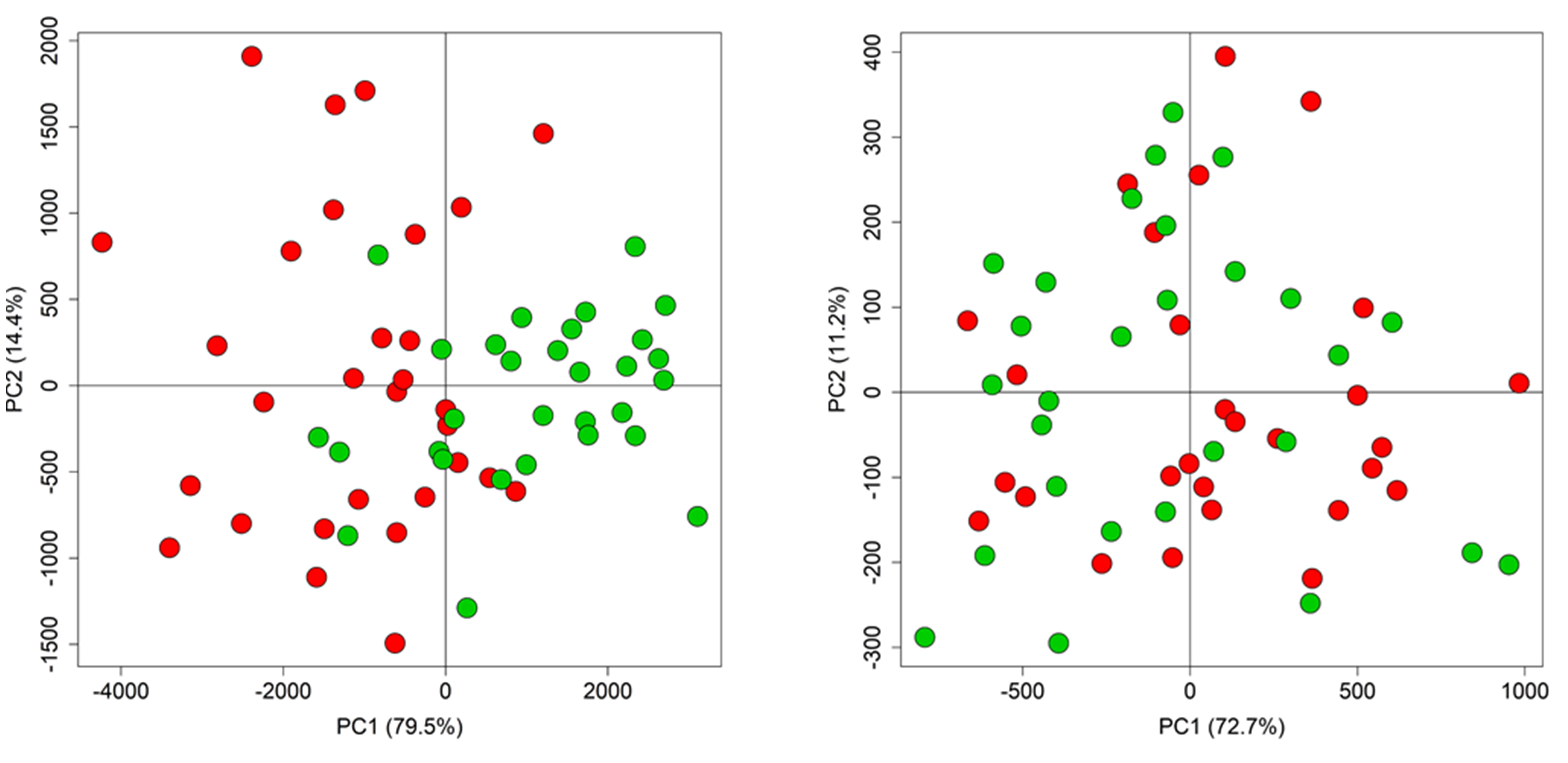

Supplement: S1 Fig — Score plots (PC1 vs. PC2) of the unsupervised PCA model of A) 21 quantified metabolites, B) lipoprotein-related parameters; COVID-19 patients (red dots); CTR subjects (green dots). (TIF) [file ppat.1009243.s001.tif]

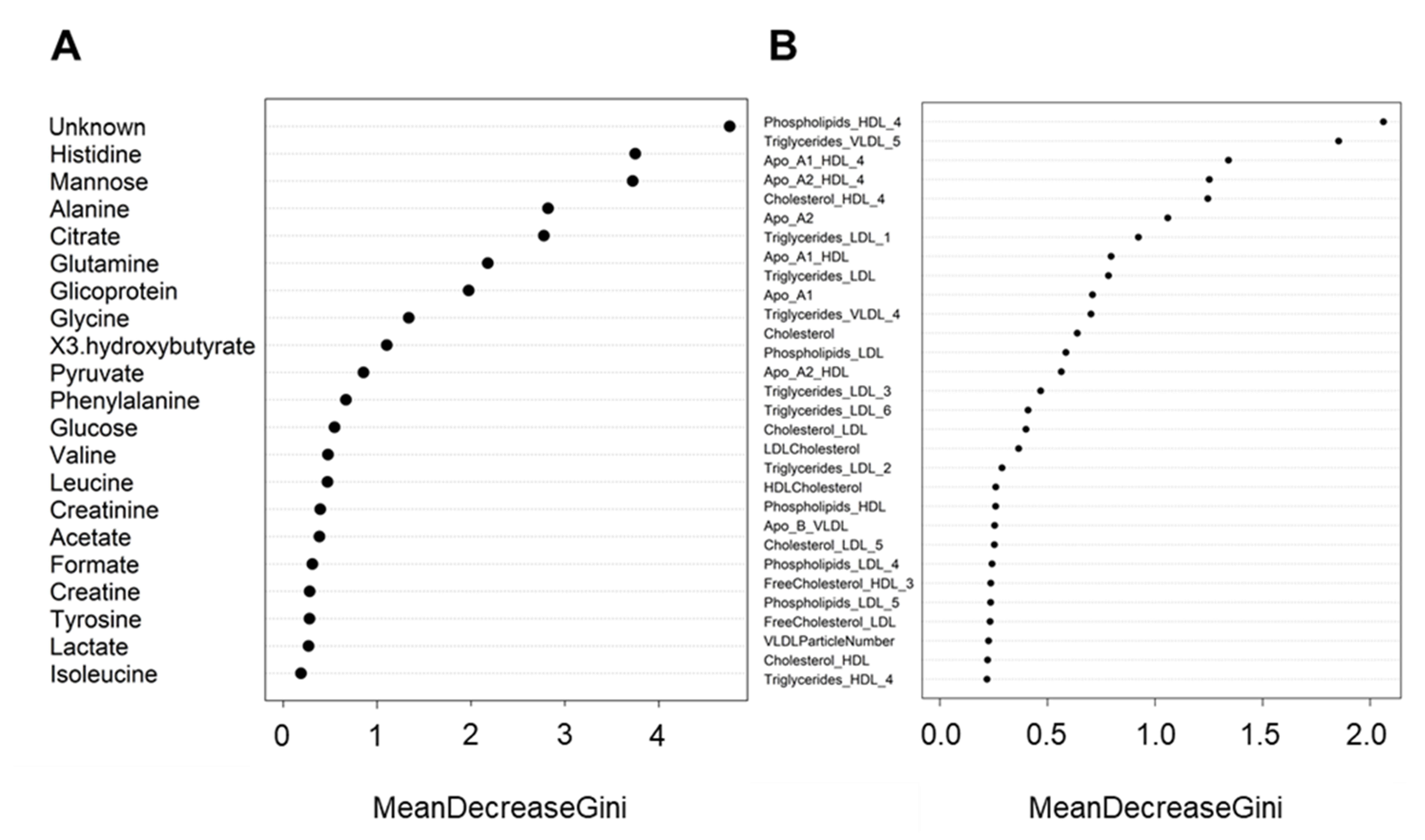

Supplement: S2 Fig — Variable importance plots of the Random Forest models discriminating COVID-19 patients and control subjects. A) 21 quantified metabolites, B) lipoprotein-related parameters. (TIF) [file ppat.1009243.s002.tif]

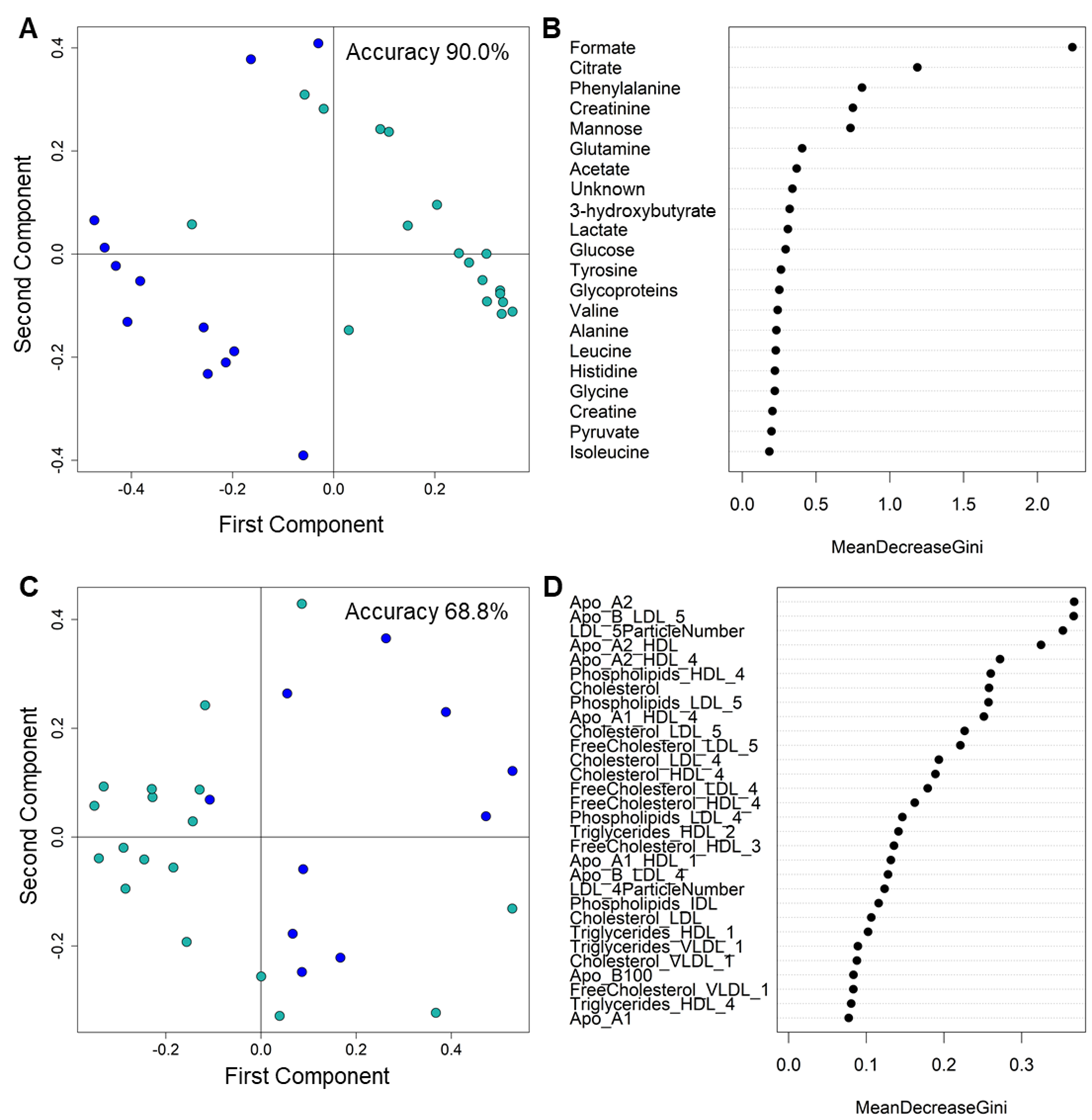

Supplement: S3 Fig — Proximity plot (of the first two principal components) and accuracy of the Random Forest model discriminating COVID-19 patients treated (blue dots) and non-treated (sea green dots) with invasive ventilation using metabolites (A) and lipoprotein-related parameters (C). Variable importance plots of the two Random Forest models: B) 21 quantified metabolites, D) lipoprotein-related parameters. (TIF) [file ppat.1009243.s003.tif]

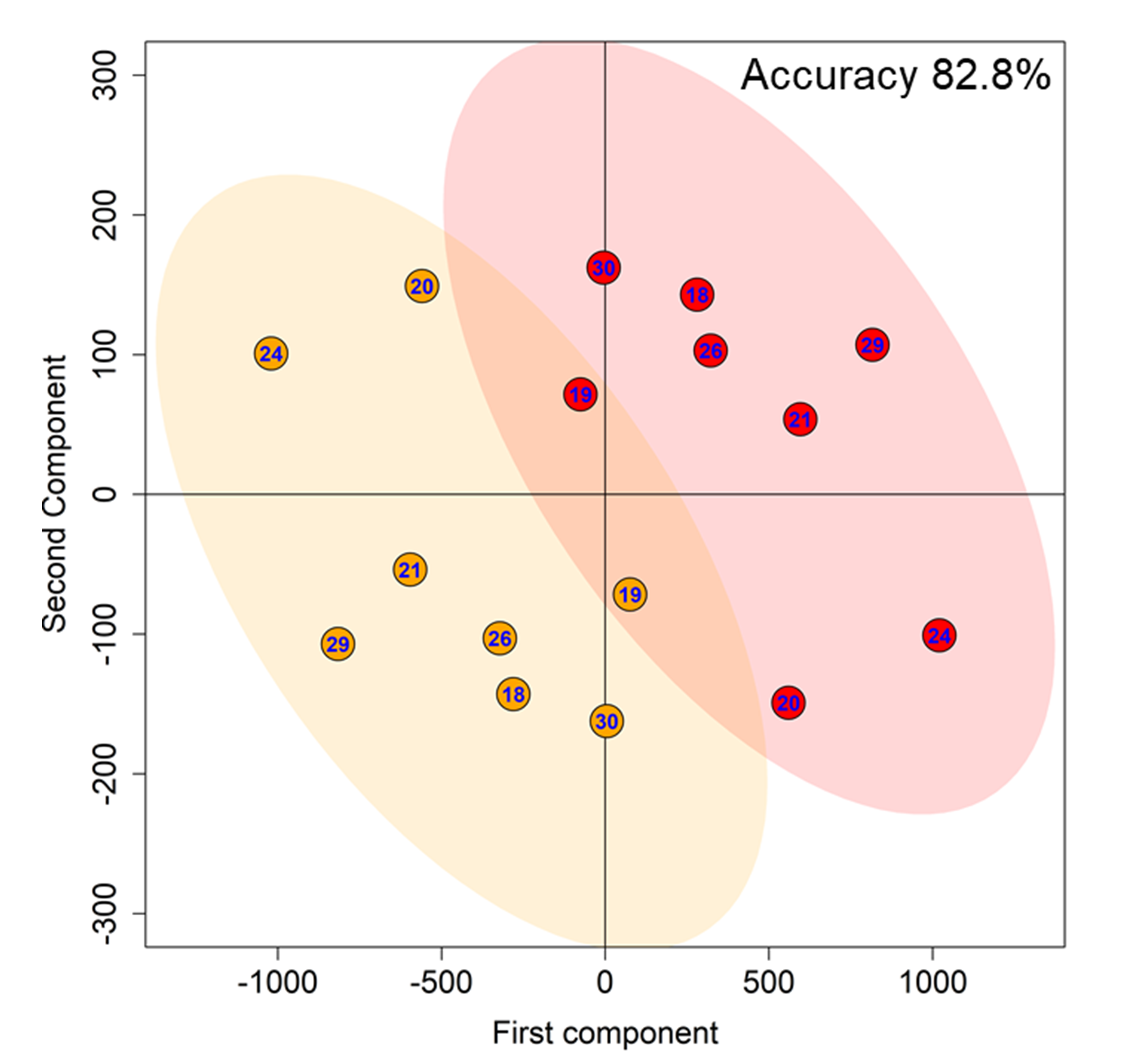

Supplement: S4 Fig — A) Score plot (of the first two principal components) and accuracy of the mPLS-DA model discriminating COVID-19 patients pre- (red dots) and post- (orange dots) tocilizumab treatment using the lipoprotein-related parameters. (TIF) [file ppat.1009243.s004.tif]
